# Analysis of Job Failure and Prediction Model for Cloud Computing Using Machine Learning

**DOI:** 10.3390/s22052035

**Published:** 2022-03-05

**Authors:** Mohammad S. Jassas, Qusay H. Mahmoud

**Affiliations:** Department of Electrical, Computer and Software Engineering, Ontario Tech University, Oshawa, ON L1G 0C5, Canada; qusay.mahmoud@ontariotechu.ca

**Keywords:** cloud computing, failure prediction, fault tolerance, Random Forest (RF), Google cluster trace, Trinity trace, Mustang trace

## Abstract

Modern applications, such as smart cities, home automation, and eHealth, demand a new approach to improve cloud application dependability and availability. Due to the enormous scope and diversity of the cloud environment, most cloud services, including hardware and software, have encountered failures. In this study, we first analyze and characterize the behaviour of failed and completed jobs using publicly accessible traces. We have designed and developed a failure prediction model to determine failed jobs before they occur. The proposed model aims to enhance resource consumption and cloud application efficiency. Based on three publicly available traces: the Google cluster, Mustang, and Trinity, we evaluate the proposed model. In addition, the traces were also subjected to various machine learning models to find the most accurate one. Our results indicate a significant correlation between unsuccessful tasks and requested resources. The evaluation results also revealed that our model has high precision, recall, and F1-score. Several solutions, such as predicting job failure, developing scheduling algorithms, changing priority policies, or limiting re-submission of tasks, can improve the reliability and availability of cloud services.

## 1. Introduction

Fault tolerance for cloud computing can provide uninterrupted cloud services, even if one or more components can be failed for any reason. Due to heterogeneity and its large-scale nature, cloud architectures have become more complex than traditional distributed systems. Thus, new modern IoT-Cloud applications, including smart cities and eHealth, require new design architectures that provide high reliability and availability. Both cloud providers and consumers are concerned about availability and reliability. The primary reason for this concern is that the cloud architecture is complex, which increases the probability of failure [1,2,3].

Cloud providers face reliability-related challenges that are dramatically similar to those encountered years ago. These challenges are power outages, unexpected hardware failures, failed deployments, software failures and human errors. The reliability and availability of cloud computing remain the main concern of cloud consumers. For example, Amazon Web Services (AWS) has experienced a failure in one of its services, Elastic Block Storage (EBS). This failure brings down thousands of hosted websites and applications for 24 h [4]. Host applications and websites require fault tolerance to overcome the effects of failure and to perform their tasks correctly when failures occur. Cloud providers are responsible for maintaining their services to provide cloud consumers with a high quality of services (QoS).

The coronavirus pandemic (COVID-19) has tested cloud providers in many ways, none of which could have been predicted. Although the public cloud has proven remarkably resilient in overcoming an unprecedented stress test, there are unusual exceptions to cloud failure problems in the first half of 2020. For example, on 3 March 2020, almost all Azure services in Microsoft’s East US region encountered storage/connectivity problems for more than six hours, starting at 9:30 a.m., according to the company’s Azure status page [5].

The analysis of failure is different from the prediction of failure. Failure analysis techniques are critical for identifying the source of hardware and software failures in an operating cloud system. The main purpose of the failure prediction, on the other hand, is to identify failed tasks before they occur. A failure analysis and prediction model is developed and implemented to identify the most critical cloud application metrics and characteristics. The fundamental goal of failure analysis and failure prediction is to investigate the frequent changes and behaviour of cloud apps in order to optimize their performance and minimize failed tasks.

A huge amount of memory and CPU resources is wasted when a job fails. Many previous studies [2,6] have examined and categorized the workload characteristics of Google traces [7]. The most recent studies have concentrated on evaluating and investigating failure behaviour, while there are limited research findings in the development of job failure prediction models [1,8,9,10].

We enhance our previous work in [11] by developing a novel generic model for failure prediction, which we have evaluated on three different workload traces and found to be effective. To the best of the knowledge found, no previous work has designed and implemented a failure prediction model applying different classifiers based on machine learning algorithms: Decision Trees (DTs), Random Forest (RF), K-Nearest Neighbours (KNN), Quadratic Discriminant Analysis (QDA), Gradient Boosting, XGBoost and Naive Bayes (NB), and applied the proposed model to three different cloud traces: Google, Trinity and Mustang. The model’s performance has been evaluated based on several criteria to ensure that the proposed model can provide high accuracy in the prediction phase.

Therefore, the main contribution of this research is as follows:

- Analyze and study various cloud traces to find the correlation between (failed and successful) jobs/tasks and cloud trace features.
- Propose a model for failure prediction that can detect failure early before it suddenly occurs.
- Design and implementation of a failure prediction model based on the use of various machine learning algorithms and three cloud workload traces.
- Evaluate our proposed model in order to ensure that the model is generic and can be applied to various cloud traces. For this reason, we have applied different ML classification algorithms to different cloud workload traces. Then, based on the evaluation results, we have selected the best model that can achieve the highest accuracy rate.

We divide the remainder of the paper into the following sections: Section 2 discusses and summarizes relevant work on failure analysis and failure prediction. Our proposed model is described in Section 3. The experiments and evaluation results of the proposed model are presented in Section 4. Section 5 provides a discussion. Finally, we conclude the work in Section 6 and offer ideas for future work.

## 2. Related Work

This section provides an overview of the literature, including a discussion and summary of relevant work on failure analysis and failure prediction.

### 2.1. Failure Analysis

Failure analysis and characterization have been extensively researched in cloud computing, grid computing, and supercomputers [1]. Certain studies place a greater emphasis on the dependability of cloud computing hardware [12]. Pan et al. [13] present the Ganesha method, a black-box diagnosis tool that uses operating system data to discover and diagnose MapReduce system issues. Zhang [14] examined the workload characterizations provided by realistic performance benchmarks in order to determine the influence of cloud system modifications on performance. A forecasting technique based on Generalized Autoregressive Conditional Heteroscedastic (GARCH) and Autoregressive Integrated Moving Average (ARIMA) models was presented by Amin et al. [15] to estimate time between failures and response time in web services. Khan et al. [16] found the repetitive workload patterns of virtual machines. They then developed a technique based on Hidden Markov Modeling to describe and forecast the workload patterns of virtual machines.

Numerous studies use Google cluster traces [7], including workload trace characterization [1], and statistical approaches to compare Google datacenters, which are classified as cloud platforms, to Grid or HPC systems [17]. Fadishei et al. [6] examine workload characteristics such as CPU speed, memory usage and utilization, task execution time, and other monitoring data. They discover a relationship between unsuccessful jobs and workload characteristics. Reiss et al. [2] have analyzed the Google cluster traces in order to demonstrate how the big-data workload is diverse and extremely dynamic. Nevertheless, this study does not include the importance of job failure analysis and prediction. Hence their work is restricted to a generalized examination of the Google cluster trace. Liu et al. [18] studied and analyzed the Google traces, including the distribution of machines. The authors have summarised statistical data relevant to tasks, jobs, and machine events. Garraghan et al. [19] analyzed the server characteristics and resource use on the Google platform using the same dataset of Google traces. Additionally, they examine the impact of jobs that are cancelled before they are properly finished, resulting in resource waste. Mesbahi et al. [20] offer a dependability study and a Markov model based on Google cluster usage traces. They have also investigated the reliability of Google cluster traces using various physical machine failure rates and characteristics, such as steady-state availability, mean time to repair, and mean time to failure. Ruan et al. [21] have introduced a comprehensive multi-view approach to comparing two cloud workloads and conducted a case study on Alibaba and Google cluster trace. Ahmed et al. [22] have utilized Google cluster workload to discover the distribution function for the time to repair and the time to failure for the cloud servers.

Some studies have used unsupervised learning in machine learning in order to characterize cloud applications based on jobs and tasks events [23,24]. Di et al. [23] have identified cloud apps based on task events and resource utilization using a K-means clustering technique with an optimal number of sets. The number of applications in the K-means clustering sets is distributed in a way that is comparable to the Pareto distribution. Alam et al. [24] conducted a statistical analysis of resource usage and workload traces. Although numerous previous studies have analyzed Google cluster traces for workload patterns, the important contributions of this study are the clustering of Google workload patterns and job categorisation using K-means clustering.

Amvrosiadis et al. [25] have introduced four novel traces, two from private clusters and two from HPC clusters. According to their findings, workloads in private clusters, which consist of data analysis tasks that are likely to be more closely linked to Google’s workload, are more similar to those in HPC clusters. This observation shows that other traces should be taken into account when evaluating the generality of new findings. Their new traces include two from Two Sigma’s private cloud and two from the high-performance computer clusters at Los Alamos National Laboratory (LANL) [26].

In [27], we examined workload characteristics such as memory utilization, CPU speed, and storage space. We observe a direct correlation between unsuccessful jobs and workload characteristics. Additionally, there is strong evidence that terminated and unsuccessful tasks utilized a sizable part of cloud resources. A small percentage of failed jobs were resubmitted several times in an attempt to complete them successfully. However, because these unsuccessful operations consumed many resources, they were classified as killed jobs. All tasks with a scheduling class of (3) failed. This issue demonstrates a direct relationship between the scheduling class and failure.

### 2.2. Failure Prediction

Zhao et al. [28] approach the topic of disk failure prediction from a completely different perspective than the previous researchers. They use different characteristics measured at successive time intervals for a disk drive as time series, and they utilize HMM and Hidden Semi-Markov Model (HSMM) to model such time series in order to identify “failed” disks from “good” disks. Morais et al. [29] developed a framework for the development of auto-scaling services based on a variety of CPU usage prediction algorithms, including Linear Regression (LR), Auto Correlation (AC) and Auto Regression Integrated Moving Average (ARIMA). Moreover, a pattern matching and state-driven technique were used to estimate workloads by Gong et al. [30] in order to build the workload prediction system called Predictive Elastic reSource Scaling (PRESS). It begins by employing signal processing techniques to determine whether or not the CPU used in a virtual machine displays recurrent activity patterns. If the answer is yes, the repeated patterns are utilized to estimate future workloads; if the answer is no, PRESS takes a statistical state-driven technique. A discrete-time Markov chain is used to predict demand for the upcoming few days or weeks.

Earlier research on job failure has mostly focused on the study and characterization of failures. However, little research has been published on the prediction of job/task failure [1,8,10,31,32,33,34]. Samak et al. [35] have applied the Naive Bayes classification algorithm to the execution logs of scientific processes to predict the failure of tasks. Then they have shown that in some situations, an incoming task that is predicted as a failed task can be successfully scheduled to a different available resource. Bala and Chana [36] proposed architecture for task failure prediction using data analysis and machine learning algorithms so that their approach can be classified under proactive fault tolerance techniques. Thus, their approach is applied during the execution time of the applications before the failure occurs. Liang et al. [31] using system data from an IBM BlueGene computer to predict failure by examining the features of fatal failure events and establishing a correlation between non-fatal and fatal events. El-Sayed et al. [8] developed a model for predicting job failure based on a Random Forest (RF) classifier. Rosa et al. [10] have proposed an approach for predicting the outcome of task events. Their methodology is based on the integration of three distinct algorithms: Quadratic Discriminant Analysis (QDA), Linear Discriminant Analysis (LDA), and Linear Regression (LR). Each day, the classification model is updated. Shetty et al. [32] have proposed a model based on the XGboost classifier that makes use of three distinct resampling strategies. They obtained an accuracy of 92% and a recall of 94.8%. Machine learning classifiers were tested to predict job failures by Hongyan et al. [37]. They evaluated the performance of four different algorithms: RF, KNN, KDT, and LR. In order to test the classifier’s accuracy, the OpenCloud dataset is used. Sun et al. [38] used a deep learning model to predict a software failure. Additionally, they used a mechanism for creating fresh samples to generate failure data. Different machine learning classifiers were used by Padmakumari and Umamakeswari [39] to predict task failure in scientific applications. Classifiers were trained and tested on a synthetic dataset. The results reveal that the NB classifier has a high rate of accuracy (up to 94.9%).

Deep learning has been used by Gao et al. [40] to predict job and task failures. They used a model called Bidirectional Long Short Term Memory (Bi-LSTM) with many layers. In order to model training and testing data sets, the authors extract static attributes and generate dynamic ones. According to the results, Bi-LSTM predicted task failure with an accuracy of up to 93% and job failure with an accuracy of up to 87%. Moreover, Islam and Manivannan [41] found important attributes related to cloud application failure. In order to predict the application’s termination state, they used the LSTM model. The results indicate that LSTM achieves up to 87% of accuracy.

One of the most significant shortcomings of previous research is that most studies evaluated their models against a single classification technique without comparing them to other classifiers to ensure that their results were accurate. As a result, we have applied various classification methods, including DTs, RF, KNN, XGBoost, NB, Gradient Boost and QDA. We then selected the ideal model based on the use of several evaluation criteria and selection methods.

A failure prediction model has been proposed in our previous work [42], and we improved the model’s accuracy by using multiple feature selection techniques. Our failure prediction model outperformed others in previous studies [8,33]. Table 1 summarizes the most current studies on the analysis and prediction of job and task failures.

## 3. Failure Prediction Model

This section discusses the main components of the proposed framework for failure analysis and prediction. The failure prediction model has been designed and implemented to predict the termination status of submitted tasks before the execution time.

**Figure 1:**
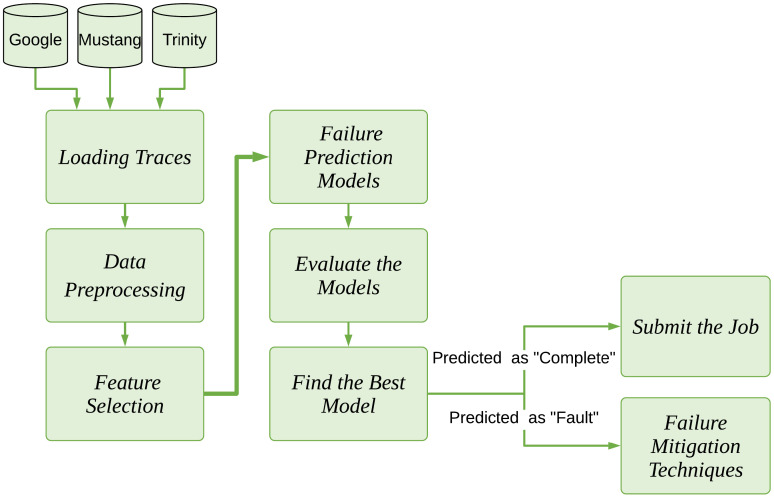
The proposed evaluation process.

Algorithm 1 describes the failure prediction model. The algorithm inputs cloud trace workload *D*, which includes a number of tasks. The algorithm also applies and tests different feature selection techniques and classifier models to the selected cloud trace. The algorithm returns the termination status “failed/finished”. Set *D* represents the dataset that is extracted from the input cloud workload trace. The data preprocessing has applied to the selected dataset. The data preprocessing steps include the cleaning and filtering out of all tasks that were submitted thousands of times. After that, the management system kills them after they have consumed many resources. The selected cloud trace is used for training and testing the prediction models. The *M* indicates the selected model used in the prediction for classifying *failed* and *finished* tasks. At the beginning of the proposed algorithm, *performanceList* and *topRankFeaturesList* are initialized with a *Null* value (line 1). The *topRankFeaturesList* is a list for storing the best features for each feature selection technique to be used as input for the selected model *M*. The algorithm then tests different feature selection techniques *(SelectKBest, Feature Importance, Recursive Feature Elimination (RFE))* with different prediction models *(RF, DS, NB, QDA, KNN, XGBoost, and Gradient boosting)*, which present in lines 2 and 3. The algorithm also considers data preprocessing as one of the most important steps before applying the proposed model (lines 4–9). The algorithm then applied different selection techniques to different prediction models (lines 10–15). Then the algorithm evaluates the performance and stores the results to the *performanceList* (line 16 and 17); after that, the algorithm selects the proposed model based on the best performance results (line 21). The algorithm predicts the termination status (line 23) of using the failure prediction model. If the task termination status is greater than (0), it is predicted to be “*failed*”, and one of the failure mitigation techniques will be applied (lines 24–26). Otherwise, the termination status of the task is predicted as “*finished*” (line 28). Additionally, the proposed evaluation process for the failure prediction model is depicted in Figure 1.
**Algorithm 1:** Failure Prediction Algorithm
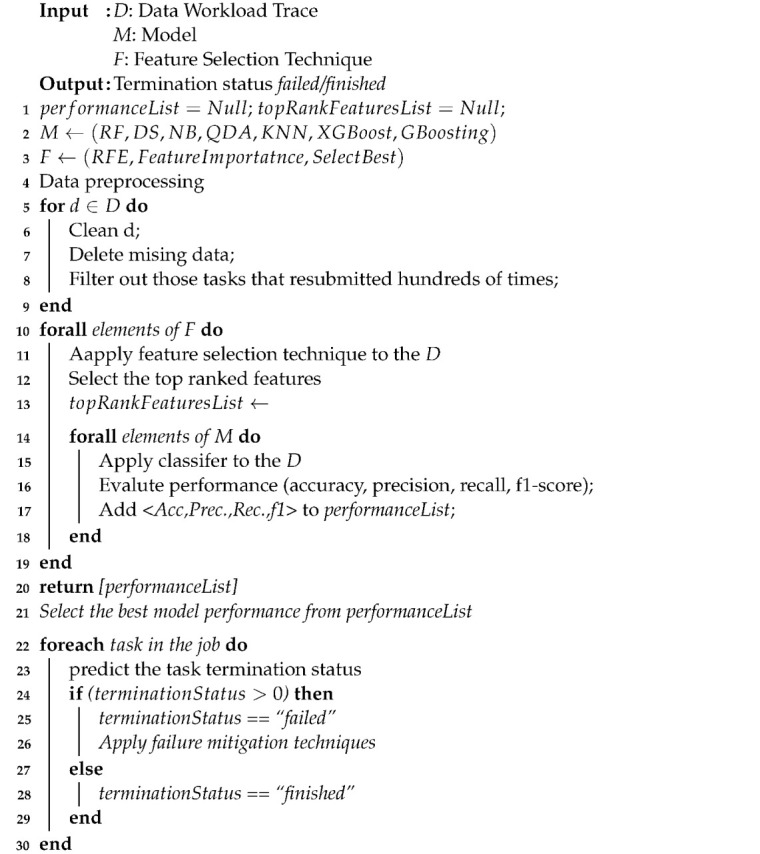


We can summarise the proposed model’s process as follows:

(1) The workload data was collected from three different traces, with the purpose of ensuring that our proposed model could be applied to traces of various lengths.
(2) To ensure that the data is prepared for classification and modelling, analysis, pre-processing, filtering techniques have been performed to the three traces.
(3) Three feature selection techniques have been applied to the traces to improve the proposed model’s accuracy and performance. After that, we can rank the most important features based on the results.
(4) To predict failed and finished jobs, four machine learning classification approaches were used to the traces.
(5) Finally, the cloud management system determines the appropriate failure prediction model based on the best prediction findings. If the job is predicted as “finished”, it will be submitted and typically scheduled to available nodes. Otherwise, failure mitigation techniques will be utilized if the incoming task predicted as “failed”, but it is important to note that this phase will be handled in future work.

This study extended and improved the prior work [11,27] on failure analysis and failure prediction models. The primary goal of our failure prediction model is to early predict “failed/finished” tasks in the cloud applications with high rate of accuracy using ML classification algorithms. The proposed model helps to reduce computational time and resource consumption, and it increases the efficiency and performance of cloud infrastructure.

### 3.1. Data Preprocessing and Filtering

The model’s input is a set of features that can describe the *job/task* attributes and the behaviour of a cloud system. We have filtered out all event types of *jobs/tasks* that have not occurred within the Google trace window (from 1 to 29 May), and these jobs and tasks are presented as a time of 0 in the trace window. We have also converted all jobs and tasks timestamp microsecond to daytime. In Google cluster trace, we investigated why the number of failed tasks is very high compared to the number of failed jobs, so we found that some tasks are resubmitted thousands of times to be successfully finished. However, these tasks were killed after they had consumed considerable resources. As a result, we have removed these tasks, which include these types of tasks, because they are considered outlier cases. During this period, we expect a data centre outage to occur.

### 3.2. Feature Selection

First, we manually select the most relevant characteristics to save training time for the classification algorithm and avoid over-fitting. Then, in order to increase the accuracy of our failure prediction model, we used feature selection algorithms including Feature Importance, SelectKBest and Recursive Feature Elimination (RFE).

### 3.3. Prediction Techniques

The Decision Trees (DTs) classifier is one of the supervised learning algorithms that we employ in our research. In order to learn from data characteristics, the DTs algorithm incorporates many decision-making principles, which are discussed more below. The entropy of a split is the criterion that we employ to evaluate its overall quality. The DTs algorithm uses entropy to determine the homogeneity (number of equivalent values). The cost complexity of a tree may be calculated using the number of leaves in the tree and the error rate of the tree. It may be necessary to employ other classification algorithms to discover the most accurate, efficient, and dynamic model solution within a reasonable situation. We utilized seven Machine Learning (ML) classification algorithms in this paper: Random Forest (RF), Decision Trees (DTs), K-Nearest Neighbours (KNN), Quadratic Discriminant Analysis (QDA), Gradient Boosting, XGBoost and Naive Bayes (NB).

## 4. Experiments and Evaluation Results

This section covers the implementation of the proposed framework and the experimental evaluation results as well as a discussion.

### 4.1. Experimental Setup

Comma Separated Value (CSV) files are used to store all used traces. Google, Mustang, and Trinity each have a trace size of around 15 GB, 280 MB, and 14 MB, respectively. Python data frames are used to store data. The failure prediction model is then implemented using scikit-learn, a python machine learning library [50]. We have performed this experiment on “Google Colab Pro+” because Google trace has a big data size that requires a high computational processing power for analysis and prediction. We used 64 GB of RAM and 256 GB of disc storage on the Google Colab Pro+ resources.

### 4.2. Traces Analysis

As researchers, we value how well prediction models work when they are tested against real-world workloads. However, anyone who has tried to find data to accomplish this kind of analysis knows that there are few available public workload traces. Legal and cultural barriers to disclosing data are often responsible for this data availability. Even when data sets are made publically available, they are presented in noncanonical forms, remove elements that are valuable to researchers, and are published separately on websites that eventually become inaccessible to researchers [26].

We analyzed and utilized three large-scale traces accumulated by a variety of entities, including Google and the Los Alamos National Laboratory (LANL). The next subsections provide a more extensive explanation of the traces utilized. Basic description and characteristics of the clusters having traces in the Atlas repository and Google cluster traces are shown in the Table 2. In comparison to other traces, the Google trace has a high failure rate. Google trace, on the other hand, has over 28 million tasks submitted, making it the most comprehensive trace accessible. The LANL Mustang trace has the longest trace time when compared to the other traces.

#### 4.2.1. Google Cluster Traces

Google has made a massive dataset called Google cluster traces publicly accessible. This dataset contains a large number of monitored data files that were created by a large system with over 12,500 nodes. There are 672,074 jobs and over 28 million tasks in the Google cluster traces submitted between 1 to 29 May. A further observation is that each task has distinct features depending on its scheduling class, priority, requested resources, and actual resource consumption. We summarize basic statistical information of Google traces in Table 3. In the following subsections, we explain the correlation between failed tasks and priority levels, scheduling classes and requested resources.

**Figure 2:**
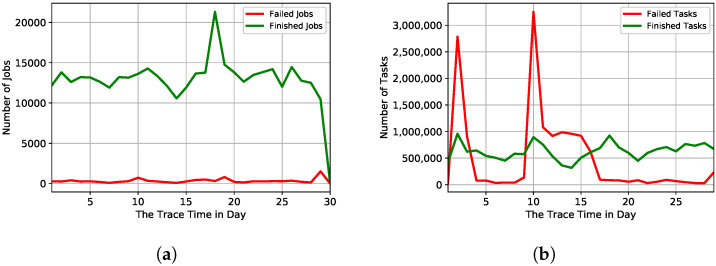
A comparison between job and task event failure behaviour in 29 days of Google trace. (**a**) Failed and finished jobs for 29 days of the Google trace; (**b**) Failed and finished tasks for 29 days of the Google trace.

**Figure 3:**
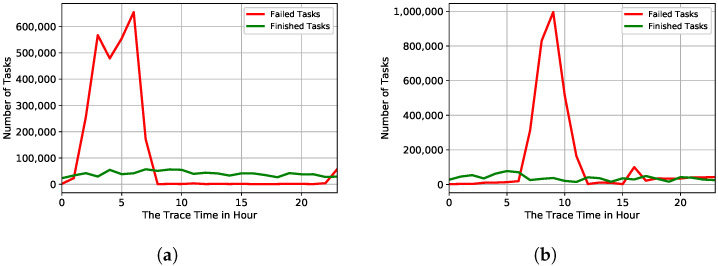
Number of failed and finished tasks for Google cluster trace, focusing on days 2 and 10. (**a**) Day 2 of the Google traces; (**b**) Day 10 of the Google traces.

In the preprocessing data phase, we investigate the failure behaviour for 29 days. Figure 2 shows a comparison between job and task event failure behaviour in 29 days of Google trace. In Figure 2a, failed and finished jobs follow normal behavior. However, as shown in Figure 2b, the number of task failures has significantly increased in the second and tenth days of Google’s trace. Figure 3 presents many failed tasks during the Google trace period, focusing on the trace’s second and tenth day. On the second day of the trace, we notice that most task failures occurred between 1 a.m. and 8 a.m as shown in Figure 3a. On the tenth day of the trace, most task failures occurred between 6 a.m. and 12 p.m. as shown in Figure 3b. Therefore, we estimate that a portion of the data centre was unavailable.

**Figure 4:**
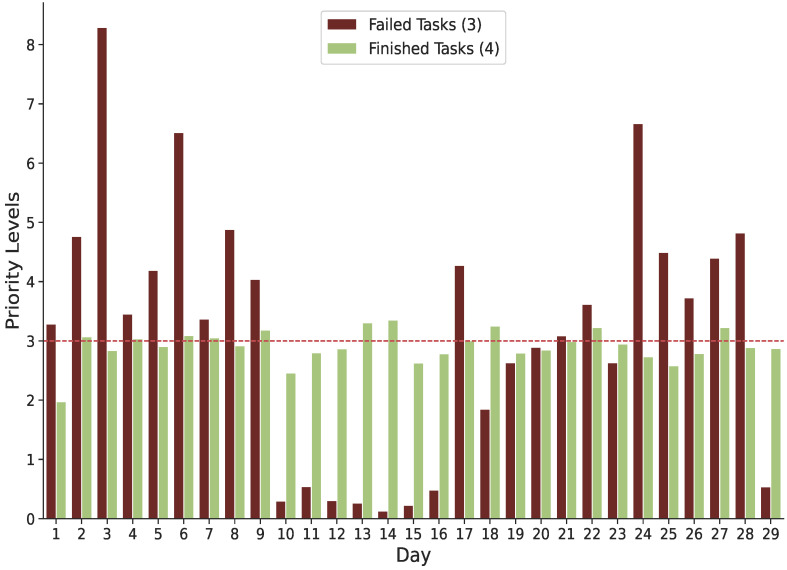
Priority level for failed and finished tasks.

**Failure and Priority Levels:** A priority level is assigned to each task, represented by a tiny number that is translated into a sorted collection of values, with (0) being the lowest priority (least significant). Tasks with higher priority numbers often receive more resources than tasks with lower priority numbers. The average priority level for failed and completed tasks during the trace period is shown in Figure 4. Our findings indicate that the most of unsuccessful tasks, notably on the third day of Google’s trace, are of a medium or high priority. With a priority level greater than 3, the likelihood of tasks failing significantly increases. As a result, a positive correlation exists between failed tasks and task priority levels. In order to increase the productivity of cloud infrastructure and applications while reducing the risk of failure, priority strategies must be developed. Nearly half of all tasks have a priority level of (0) out of (9), and 28.4% have a medium priority level of (4).

Furthermore, resource requests show how much CPU, memory, and disk space a task consumes. Tasks that consume more resources than their allocated amount may be killed or terminated. Even though each job is using less than its limit, it is possible that there are not enough resources to meet all of the task’s runtime needs. This might result in the termination of one or more lower-priority tasks.

**Figure 5:**
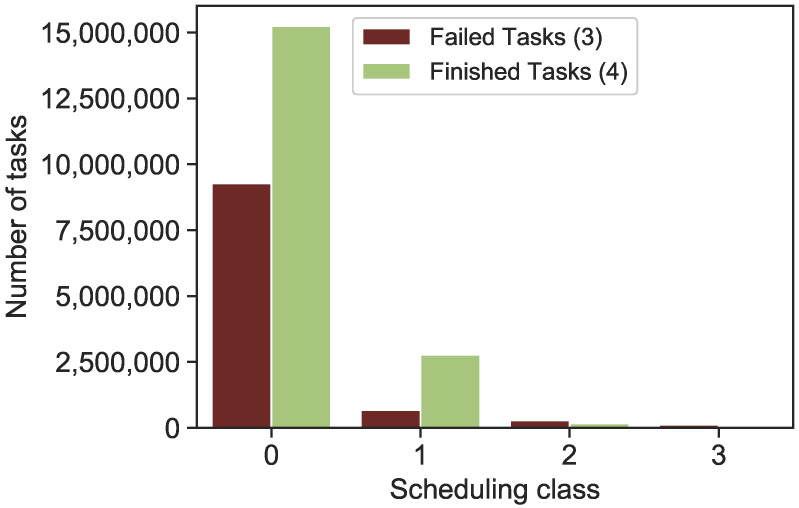
Scheduling class for failed and finished tasks.

**Failure and Scheduling Class:** All jobs and tasks are assigned a scheduling class that approximately corresponds to how latency-sensitive they are. A single number expresses the scheduling class, with 3 denoting a task that is more latency-sensitive. The relationship between the scheduling class and termination status *(failed tasks or finished tasks)* is depicted in Figure 5. Only 1% of all receiving tasks have a scheduling class of (3), whereas 73% and 18% have scheduling classes of (0) and (1). Failed or finished tasks do not appear to have a direct correlation with lower class scheduling. However, the majority of completed and unsuccessful tasks fall into the lower scheduling class (0,1), whereas a minor proportion of completed and unsuccessful tasks fall into the medium scheduling class (2). All tasks that have been received and have a type of high scheduling class (3) have been marked as failed. As an outcome, a significant correlation between unsuccessful tasks and a high scheduling class (3). As a result, a scheduling method is required to maximize the distributed efficiency of incoming tasks in terms of system availability and computation requirements.

The primary aim of the requested resource is to present the maximum amount of CPU, memory and disk space allowed. Therefore, all tasks should not exceed their limits. If some tasks exceed their limits, the management system can kill these tasks as they use more resources, including memory. In some cases, after submitting tasks to be processed, these tasks do not find enough resources to be completed successfully, even if they do not exceed their limits. Therefore, one or more tasks with low priority can be killed [7].

**Figure 6:**
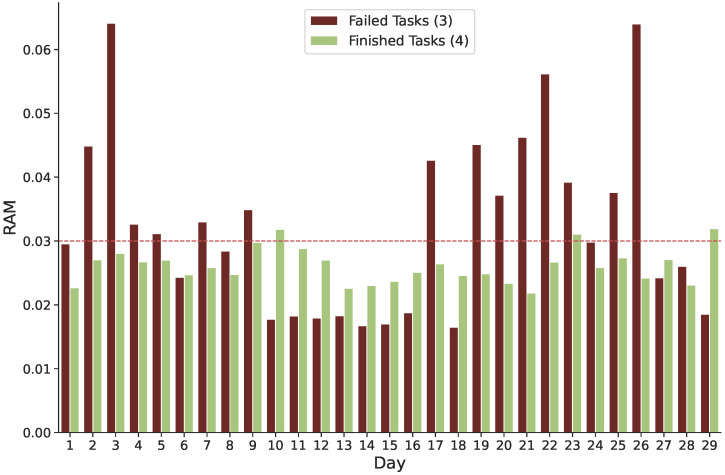
Memory was requested for both unsuccessful and finished tasks.

**Failure and Requested Memory:** As illustrated in Figure 6, there is a clear relationship between task failure and the quantity of memory required. For example, on day 3 of the Google trace, the number of failed tasks has significantly grown, and we can see that the majority of these tasks consumed more memory than the succeeded ones. When incoming tasks require a medium or large quantity of memory, the probability of failure increases. When the RAM demand exceeds 0.03, most of the tasks fail. In most cases, tasks are successfully completed when the requested memory is less than 0.03. Scheduling algorithms need to consider the required amount of computation for each task to distribute incoming tasks to the right level of resource availability. As a result of this strategy, the number of unsuccessful tasks is reduced, resulting in enhanced cloud application availability.

**Figure 7:**
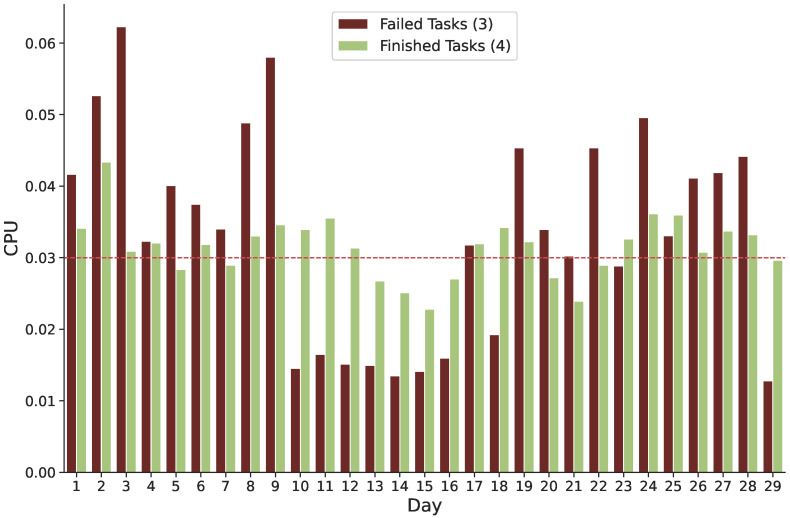
CPU was requested for both unsuccessful and finished tasks.

**Failure and Requested CPU:** A significant correlation is also discovered between task failure and the amount of CPU that was requested. Figure 7 depicts the amount of time the CPU has requested to finish the majority of unsuccessful and completed tasks. Most tasks fail when the requested CPU is greater than 0.03. On the other hand, finished tasks occur when the desired CPU is less than 0.03. The failed tasks have increased considerably on days two and three of the trace, similar to the case with the requested memory. The unsuccessful tasks require more memory and processing power than successfully completed tasks. To summarize, by enhancing the current scheduling strategy, we expect to increase cloud application availability while also reducing resource consumption on the cloud infrastructure.

**Figure 8:**
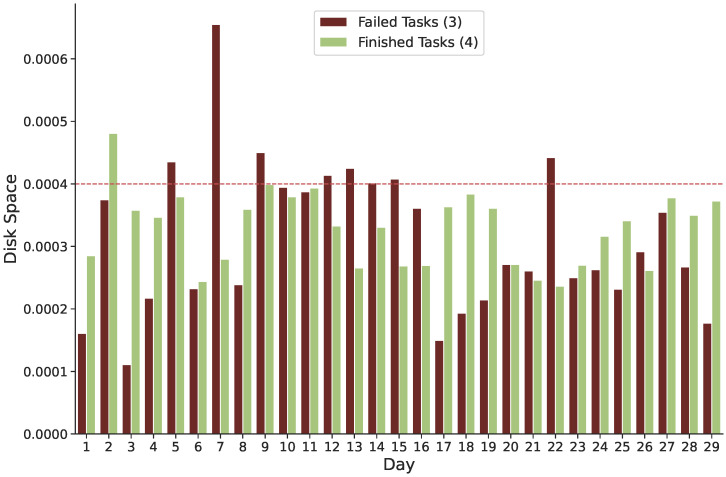
Disk space was requested for both unsuccessful and finished tasks.

**Failure and Requested Disk Space:** Failed tasks and requested disk space seem to have no apparent correlation, except on day seven of Google trace, when a significant rise in unsuccessful tasks occurs, when the requested disk space exceeds 0.0006, as illustrated in Figure 8.

In this study, we investigate the behaviour of unsuccessful tasks and the relationship between unsuccessful tasks and other factors, including task scheduling and task priority. The analysis of task and job failures can be a starting point for developing and implementing a failure prediction model that can predict the types of cloud job events (fail/finish) in advance. Cloud providers can employ novel solutions to limit unsuccessful jobs and tasks. Cloud service providers, for example, should optimize a scheduler technique to maximize the efficiency of a cloud load balancer. The load balancer plays an essential role in distributing incoming jobs and tasks based entirely on the required computation and available resources. As a result, failed tasks and jobs have been minimized, as all tasks and jobs are allocated to the appropriate resources, regardless of resource availability, and all resources are used effectively and efficiently.

Furthermore, we discovered that unsuccessful jobs and tasks run significantly slower and require considerably more resources than completed ones. Therefore, considerable resources have been spent on incomplete jobs and tasks. The development of a model for predicting early failure could help minimize the number of unsuccessful tasks and jobs. We have noticed that unsuccessful jobs and tasks have many resubmissions. In order to optimize cloud resources, it is recommended to minimize resubmissions. High-priority jobs and tasks are more likely to fail; as a result, when designing a fault prediction model, the relationship between job priority and job failure should be considered.

#### 4.2.2. LANL Mustang Cluster

The Mustang trace was collected from October 2011 to November 2016 using HPC clusters at the LANL for capacity computing. As a result, the Mustang trace can be considered to be the longest publicly available trace to date. There are 1600 compute nodes in the Mustang trace, each with 102 TB of RAM and 38,400 AMD Opteron 6176 2.3 GHz cores. Software developers, engineers and scientists at LANL primarily used the Mustang cluster. There are also 2.1 million jobs on the trace, with 565 users submitting them. The collected data include the start and finish times of the job and the type of job event, such as completed, cancelled and failed jobs [25,26].

**Figure 9:**
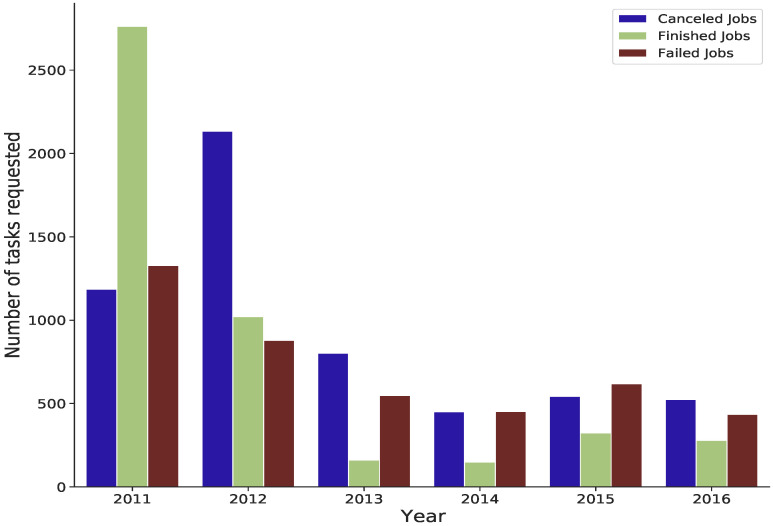
The average number of tasks requested from 2011 to 2016 for cancelled, failed and finished jobs.

A positive correlation is also found between failed jobs and the number of tasks requested for each job. Figure 9 shows the average number of tasks requested between 2011 and 2016 for cancelled, failed and finished jobs.

Most failed and cancelled jobs occurred when the average requested tasks were approximately over 500 tasks. In contrast, on average, tasks have been completed when the number of requested tasks is approximately 400 or less, particularly from 2013 to 2016.

**Figure 10:**
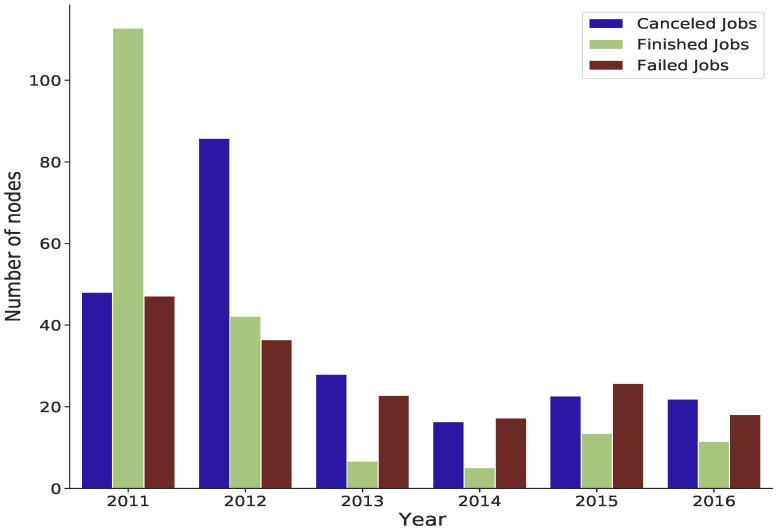
The average number of nodes from 2011 to 2016 for cancelled, failed and finished jobs.

Additionally, there is a strong association between failed jobs and the number of nodes assigned to each job. Figure 10 shows the average number of nodes for cancelled, failed and finished jobs between 2011 and 2016. Most failed and cancelled jobs occurred when the number of nodes was approximately over 18. In most cases, completed tasks occur when the average number of nodes is about 9, especially from 2013 to 2016. Between 2011 and 2016, Figure 11 shows the average number of nodes for cancelled, failed, and finished jobs in month intervals.

**Figure 12:**
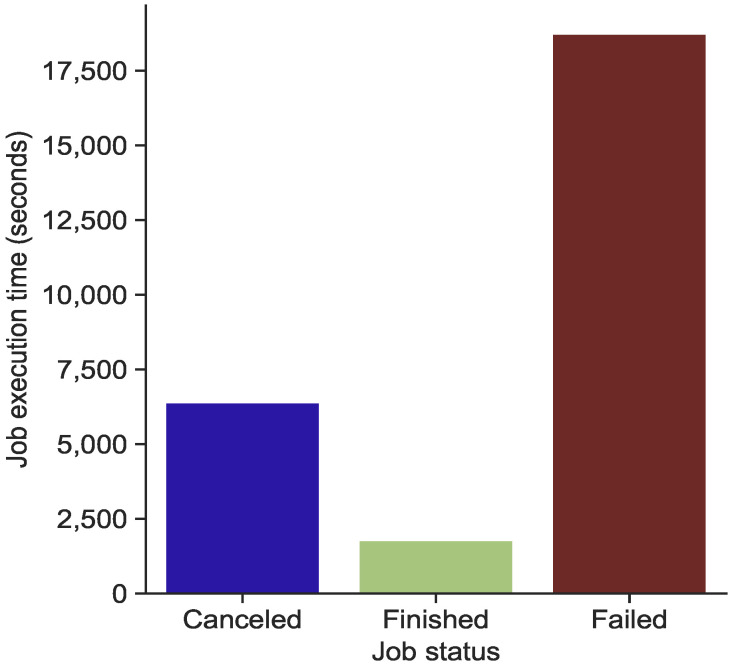
Correlation between the execution time and the failed, cancelled and finished jobs.

There is also a significant correlation between the execution time and the number of failed, cancelled and finished jobs. As shown in Figure 12, the majority of failed jobs occurred when the average execution time exceeded 17,500 s. However, most finished jobs were successfully completed, with a short execution time of less than 2500 s.

#### 4.2.3. LANL Trinity Supercomputer

Trinity is the largest supercomputer at the Los Alamos National Laboratory (LANL) and is utilized for capability computing. Trinity trace was generated using data from 9408 computing nodes equipped with 301,056 Intel Xeon E5-2698v3 2.3 GHz processors and 1.2 PB RAM, which is currently the largest cluster with a publicly released trace in terms of the number of CPU cores. This data collection spans three months in 2017, between February and April. In Trinity, there are 25,237 multi-node jobs, which are generated by 88 users [25,26].

**Figure 13:**
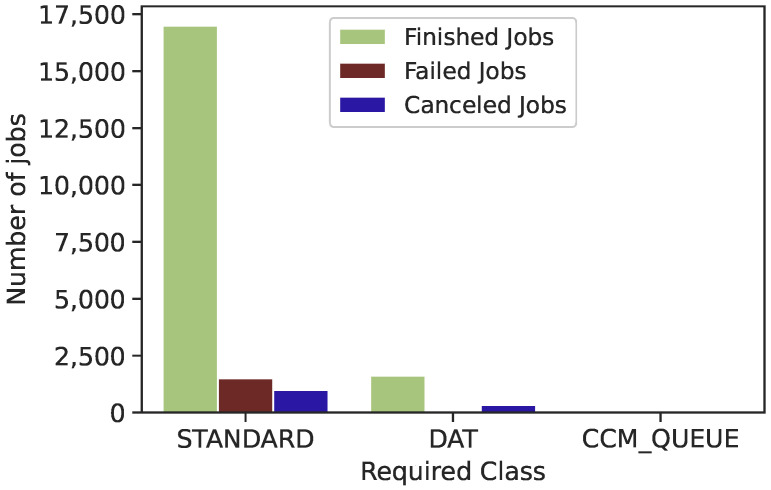
Correlation between Trinity required class types and job status.

As shown in Figure 13, Trinity trace has three categories of required class: “STANDARD”, “DAT” and “CCM_QUEUE”. Only 9% of submitted jobs are classified as “DAT” and less than 1% as “CCM_QUEUE”, compared to more than 60% of submitted jobs classified as “STANDARD”. We have found that most failed jobs are classified as “STANDARD”. However, there is no failed job classified as “CCM_QUEUE”. As a result, there is a high correlation between failed jobs and the required class attribute of the submitted jobs in the LANL Trinity trace. Hence, future work should consider designing and implementing a load balancer responsible for transferring incoming tasks based on the required class.

**Figure 14:**
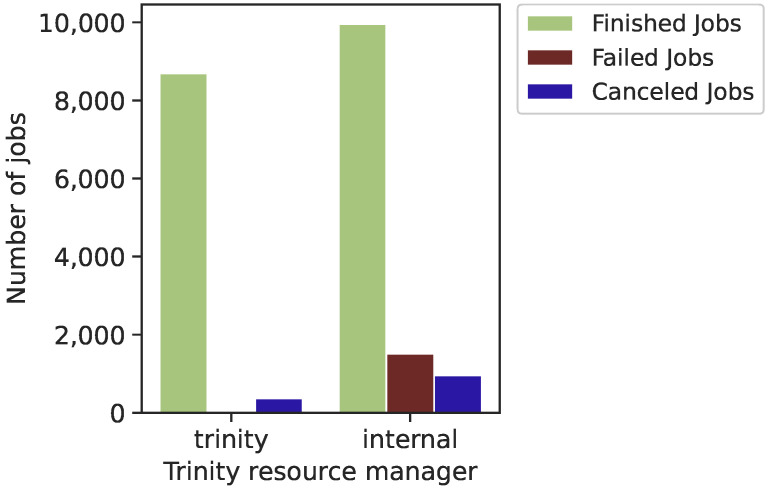
Correlation between Trinity computing resource types and job status.

As presented in Figure 14, Trinity trace contains two distinct categories of resources: “trinity” and “internal”. Approximately 60% of submitted jobs were scheduled to utilize “internal” resources, whereas roughly 40% were scheduled using “trinity” resources. We have found that most failed jobs are scheduled in internal resources. There is a high correlation between failed jobs and the attribute of the resource manager. We expected that one or more components could fail due to a failure of a network, hardware or software component.

### 4.3. Failure Analysis and Characterization

**Figure 15:**
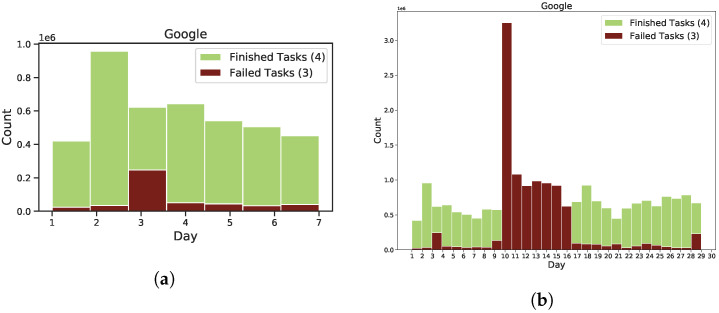
Distribution of task status for Google traces. (**a**) Google trace in 7 days; (**b**) Google trace in 29 days.

We utilized all tasks provided during the 29-day Google trace, which includes 500 CSV files. All job and task events that occurred outside of the trace window have been removed from our analysis trace. This section aims to examine failure behaviour by concentrating on the most significant events (“fail” and “finish”), which are represented as “3”, and “4” in the Google trace, respectively. Figure 15a depicts the Google trace distribution for failed and completed tasks over seven days. The first two days of Google trace show that 97% of tasks are successfully completed, whereas about 3% fail. However, between the third and fourth days of the trace, the number of completed tasks dropped to roughly 71%, while unsuccessful tasks rose to 29%. Figure 15b depicts the Google trace distribution for failed and completed tasks over 29 days. The total number of failed tasks increased dramatically between the eighth and sixteenth days.

**Figure 16:**
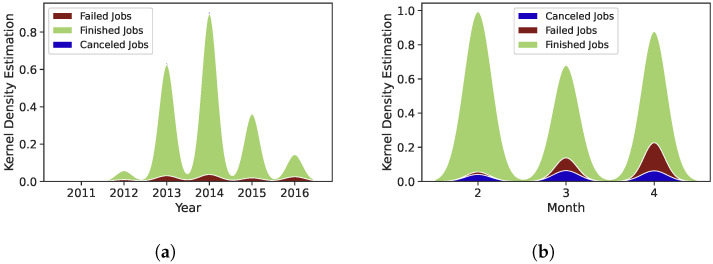
Distribution of job status for Mustang and Trinity traces. (**a**) Mustang trace; (**b**) Trinity trace.

Figure 16a shows the distribution of Mustang trace for failed and finished tasks. The number of tasks submitted in 2011 and 2012 is very low. However, the number of tasks submitted has increased from the end of 2012 to the first three months of 2014. We also noticed that the number of completed tasks has increased significantly between the last four months of 2013 and the first three months of 2014.

Figure 16b depicts the distribution of the Trinity trace for successful and unsuccessful tasks. For the second, third, and fourth months, the Trinity trace received 8601, 5699, and 7203 jobs, respectively. Thus, the submitted jobs in the second and fourth months are high compared to the third month. However, the failure rate is approximately 15% for the fourth month, which is very high compared to the failure rate in the months two and three 1% and 8%, respectively. The overall failure rate is 7.2%, and almost 65% of failure happens in month four of the trace.

### 4.4. Evaluation Metrics

To assess the feasibility of our proposed model, we evaluated its accuracy performance using several evaluation metrics [51], including accuracy, precision, recall and F1 score. One of the methods used to determine the effectiveness of the classifier model is a confusion matrix. The columns of the matrix represent observations within an actual class, whereas the rows represent observations within a target class. True Negative (TN) and True Positive (TP) samples are made for the correct prediction results. Because False Negative (FN) and False Positive (FP) are observations of inaccurate prediction outcomes, we are interested in decreasing FN and FP to improve the proposed model accuracy.

### 4.5. Classifiers and Prediction Techniques

The aim is to build a prediction model that can predict the value of the target variables after preprocessing the datasets. Several ML classification algorithms can be applied to the cloud workload traces. However, we are interested in finding the most accurate classifiers, which can be learned with a limited amount of training data.

For Google trace, our proposed model [11] has achieved high accuracy in predicting failed jobs using DTs and RF algorithms. However, our proposed model should be generic and applicable to various datasets. Therefore, we are interested in applying various classification algorithms to three different traces in order to select the best model with the highest accuracy. In this section, some classification algorithms are examined, which can be applied and evaluated with different traces. The performance of each algorithm is then assessed using multiple evaluation metrics. We separated the Google, Mustang, and Trinity traces into two parts: a training set that had 75% of the data and a testing set that contained 25% of the data.

**Figure 17:**
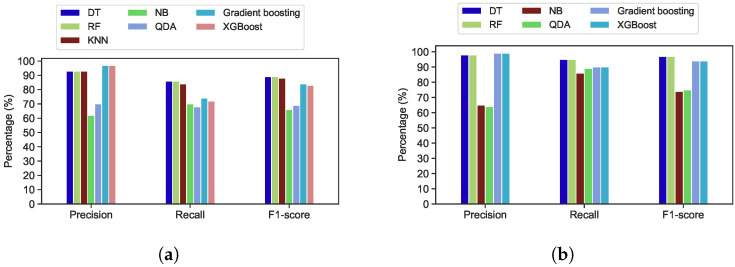
Performance evaluation of different algorithms applied to the Google trace. (**a**) Google trace in 7 days; (**b**) Google trace in 29 days.

Figure 17a depicts the evaluation results of various classification algorithms when applied to the first week of the Google trace. Precision, recall, and F1-score accuracy for both the RF and DT classifiers are 93%, 86%, and 89%, respectively. However, the results of the evaluation metrics have increased to 98%, 95% and 97% respectively, after applying DTs and RF to all Google cluster trace observations (one-month period of trace) as shown in Figure 17b.

**Figure 18:**
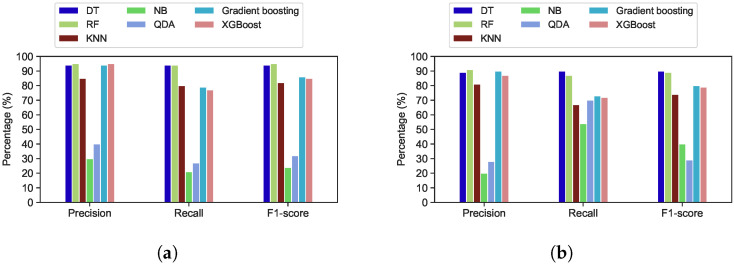
Performance evaluation of different machine learning algorithms applied to the Mustang and Trinity traces. (**a**) Mustang trace; (**b**) Trinity trace.

The same classifiers were applied for all Trinity and Mustang trace observations, as presented in Figure 18. RF and DTs have the highest accuracy when compared to other machine learning classification algorithms. The precision, recall, and f1 score of the DTs algorithms for Mustang trace are 94%, 94%, and 94%, respectively. The RF algorithms’ precision, recall, and F1-score accuracy are 95%, 94%, and 95%, respectively. In addition, compared to other models, the RF model has attained the best accuracy for Mustang, which is classified as a medium-sized trace, and Trinity, which is considered as a small-sized trace.

For Trinity trace, the number of trace observations is 20,277, which is lower than the number of observations for other traces. Therefore, all classifiers in Trinity have achieved lower accuracy than the other traces. The DTs algorithm has precision, recall, and F1-score accuracy of 88%, 89%, and 89%, respectively. The RF has precision, recall, and F1-score accuracy of 89%, 88%, and 89%, respectively. KNN, XGBoot, and Gradient Boosting have performed well compared to NB and QDA, which have the lowest evaluation metrics for all traces. However, DTs and RF classifier has achieved the highest accuracy compared to other classifiers. Table 4 shows the training and testing time of Google, Mustang and Trinity traces for utilized classifiers.

The results show that the DTs and RF can reach the highest accuracy, precision, recall, and F1-score. On the other hand, the RF-based model has longer time, at 247.6 s, with a Google trace of 29 days, while DT has only 53.8 s. Thus, DT has less complexity than RF. We attempted to apply KNN to all observations of Google cluster trace, but the processing time took more than 24 h then the memory error occurred. Thus, even though the KNN ranks third in terms of all accuracy evaluation metrics, KNN is not recommended for failure prediction due to its log processing time.

**Figure 19:**
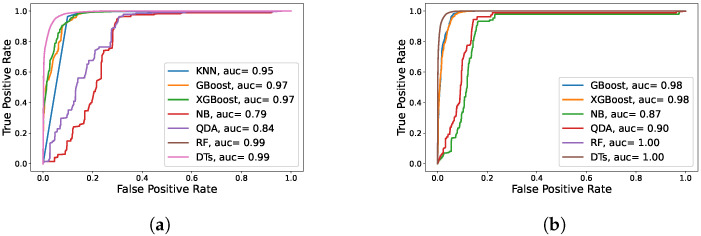
ROC evaluation of different ML algorithms on the Google cluster trace. (**a**) ROC for the first week of Google trace; (**b**) ROC for all Google trace observations.

Figure 19 depicts the Receiver Operating Characteristic (ROC) curves of the various machine learning models for the first week of the Google trace compared to one month of the Google trace. When all Google trace observations are considered, DTs and RF have reached the maximum AUC (Area Under Curve) with a high score of 100%, as shown in Figure 19b. Gradient Boosting and XGBoost, on the other hand, come in second place with 98%.

**Figure 20:**
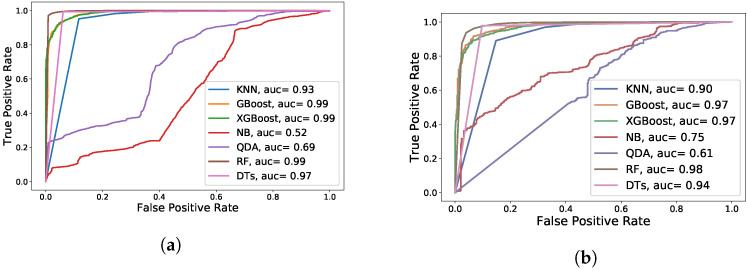
ROC evaluation of different ML algorithms on the Mustang and Trinity traces. (**a**) ROC for Mustang trace; (**b**) ROC for Trinity trace.

Figure 20 depicts the ROC curves of the various machine learning models for the Mustang trace compared to the Trinity trace. RF has reached the maximum AUC for Mustang and Trinity with a high score of 99% and 98%, respectively, as shown in Figure 20a,b. In the following part, we will discuss how to increase the accuracy of these two classifiers, RF and DTs, by using additional methods, such as feature selection.

### 4.6. Feature Selection Algorithms

Feature selection is one of the most important methods to improve the accuracy of our model by automatically selecting several features that contribute significantly to model performance. The predictive accuracy model will be reduced if the model has been trained based on irrelevant features. There are some other advantages of using feature selection, such as reducing overfitting and training time.

#### 4.6.1. SelectKBest

The SelectKBest technique uses statistical analyses to select attributes with the closest relationship to the target variable. We obtained the most important features after using this technique for Google trace: job ID, task index, machine ID, RAM, CPU, priority, and scheduling class.

#### 4.6.2. Feature Importance

Bagged decision trees, such as RF, can be used to estimate which features contribute the most and can then be used as input to a failure prediction model to predict failure accurately. We obtained the most important features after applying this technique to Google trace: job ID, day, machine ID, disk space, RAM, CPU, and scheduling class.

#### 4.6.3. Recursive Feature Elimination

One of the most well-known feature selection techniques is recursive feature elimination (RFE). The role of the RFE algorithm is to eliminate features one at a time and create a model based on the features that are left over after each elimination. RFE identifies the most significant features that can be used to predict the desired target (fail class) based on the model accuracy. For example, after we applied the RFE feature algorithm to all the attributes of the Google trace, we extracted the most important features: job ID, task index, hour, CPU, RAM, and machine ID.

Table 5 presents the evaluation results for the various feature selection algorithms used. The DTs and RF classifiers used the RFE technique to achieve the highest precision, recall and F1-score to predict failed classes, which were 99%. As a result, feature selection algorithms can enhance the proposed model evaluation metrics, including precision, recall, and F1-score. When we combined the feature importance algorithm as a feature selection technique and the RF classification algorithm as a prediction model, we were able to achieve the greatest accuracy for the Trinity trace. The highest evaluation metrics for Trinity are precision, recall, and F1-score are 90%, 88%, and 90%, respectively. Table 5 is adapted from our prior study in [42].

## 5. Discussion

This section discusses our findings and evaluation results to investigate and analyze failure behaviour for three different traces: Google cluster, Mustang and Trinity. The analysis results show the behaviour of failed and finished jobs to understand the correlation between failed jobs and other cloud application attributes. We have studied job failure as a starting point, which helps develop a new model for failure prediction. The primary goal of our failure prediction model is to predict failed tasks in cloud applications with a high accuracy rate using ML classification algorithms. The proposed model reduces computational time and resource consumption, and increases the efficiency and performance of cloud infrastructure.

As a result of our findings, we notice that failed jobs with long-running times consume considerable cloud resources compared to finished jobs. Another point regarding the job’s priority is that high-priority jobs are likely to fail. Therefore, the correlation between job failure and job priority should be taken into account in the development of a failure prediction model. Cloud providers can develop new policies for cloud applications based on our failure analysis results to decrease unsuccessful tasks and jobs. For instance, new scheduling algorithms can be applied to increase availability and reliability. Instead of transferring incoming jobs or tasks using the Round Robin technique, the incoming tasks can be transferred based on the required level of availability and computation. This technique will be considered in future work.

We found that Decision Trees (DTs) and Random Forest (RF) provided accurate results after evaluating the performance of various classification algorithms using three cloud traces. However, when it comes to the traces of Trinity and Mustang, the RF classifier surpasses the DTs. Therefore, our selected model is based on the RF method, as it applies to a wide range of traces and provides the most accurate results. El-Sayed et al. [8] developed a RF-based failure prediction model, and their findings show that the presented model can accurately recall up to 94% of all unsuccessful jobs with an accuracy of at least 95%. Compared to previous studies, we have the highest precision, recall and F1-score. Our proposed model has achieved the best precision, recall, and F1-score for predicting failed classes, which was 99% for all evaluated metrics. However, due to the small number of observations in Mustang and Trinity, we could not obtain the desired results in the other two traces. Thus, the classifier does not have enough data to distinguish between the two classes. The failure prediction model is trained and tested offline, as it requires a long training time and a high computation power. The workload traces used in this research were collected from the cloud and high-performance computing environments, such as Google cluster traces. Monitoring data indicate cloud characteristics, such as the resources required to perform a task (CPU, memory, disk storage). As a result, the proposed failure prediction model has been designed and implemented for cloud deployment. The task failure prediction model could be adapted to various forms of computing, such as edge, fog or local servers, because the observed data is similar.

## 6. Conclusions and Future Work

Failed tasks waste considerable resources in cloud clusters, including computing time, CPU, RAM, and disk space. The failure analysis describes the behaviour of failed and finished jobs in order to determine the relationship between failed jobs and other cloud application attributes. Our objective is to predict unsuccessful tasks in advance and reduce the waste associated with failed tasks. The proposed model can be used in large data centers or cloud computing environments to identify failed jobs before the cloud management system schedules them. The advantages of designing and implementing a failure prediction model include improved performance and lower costs for using the cloud. As a result, we used various classification algorithms applied to different workload traces to create a new general model that can accurately predict failed jobs with a high accuracy rate. We have also used various feature selection methods to improve the failure prediction model’s accuracy. Finally, we achieved high accuracy for Google trace by using RF and DTs classifiers as well as RFE feature selection; precision, recall, and F1-score accuracy are all 99%. In future work, we interested in applying deep learning models to publicly available traces. Furthermore, future studies will examine mitigation strategies and techniques.

## Figures and Tables

**Figure 11:**
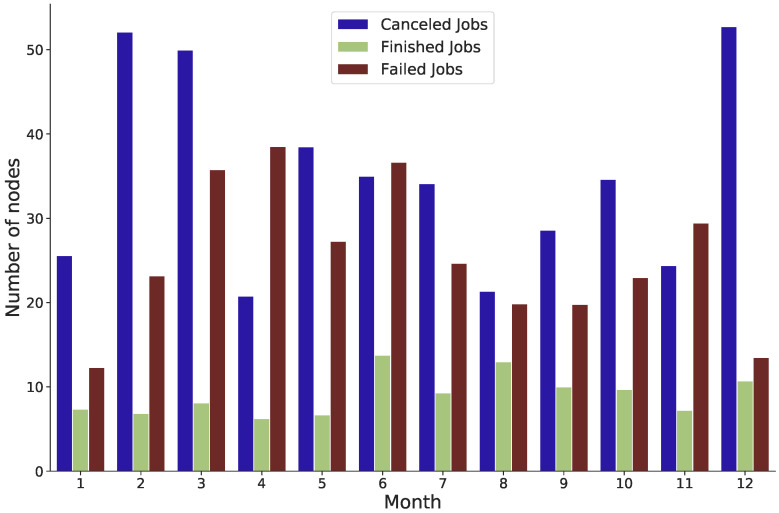
The average number of nodes for cancelled, failed and finished jobs in the month intervals between 2011 and 2016.

**Table 1:** The state of the art in the field of cloud computing for failure analysis and prediction.

References	Trace	Focus	Model	Results
Chen Xin et al. [43]	Google trace	Predicting job failure	(RNNs)	Accuracy (82%) & Recall (86%)
Garraghan et al. [44]	Google trace	Study server characteristics and resource utilization	Statistical Analysis	X
Pan et al. [13]	Google’s MapReduce [45]	Identify and diagnose faults in MapReduce	Ganesha’s diagnosis algorithm	X
Chen Xin et al. [1]	Google trace	Study characteristics of failed and killed jobs	Statistical Analysis	X
Di Sheng et al. [23]	Google trace	Understanding characteristics of cloud applications and classifying cloud applications	K-means	X
Lu et al. [46]	Alibaba	Understand machine characteristics and workload behaviors	Statistical Analysis	X
Liang et al. [31]	Log files from IBM BlueGene machine	Predict failure based on investigating the characteristics of fatal failure events	Bursty nature of failure occurrence, spatial skewness, and preceding non-fatal events	X
Zhang et al. [14]	Google’s Compute Clusters	Study the problem of deriving characterization models for task usage shapes in Google’s compute cloud.	Statistical Analysis	X
Di Martino et al. [47]	Cloud data	Analyzing causes of SLA violations for SaaS platform	Statistical Analysis	X
Chen Weiwei et al. [48]	Scientific workflow applications	Transient failures and evaluation of fault-tolerant task clustering techniques	Statistical and Probabilistic Analysis	X
Samak et al. [35]	Scientific workflow applications	Predicting failure probability	Machine Learning (Naive Bayes)	For Epigenome app Accuracy (96%)
Bala and Chana [36]	Scientific workflow applications	Task failure prediction	Machine Learning (NB RF, LR, ANN)	NB is the highest accuracy (93%)
Rosa et al. [10]	Google trace	Failure Prediction for failed tasks	Machine Learning (LDA, QDA, LR, SVM, ANN)	For ANN, accuracy (76.8%)
Liu et al. [9]	Google trace	Predicting job termination status	Machine Learning (OS-ELM)	Accuracy (93.07%) Precision (93.32%)
Reiss and et al. [2]	Google trace	Highlighting heterogeneous and highly dynamic behavior of the big-data trace	Statistical Analysis	X
Jassas and Mahmoud [27]	Google trace	Study workload features memory usage, CPU speed	Statistical Analysis	X
Wang et al. [49]	Network logs Equipment failure data	Focus on risk-aware models in optical networks and investigate how to predict risk of equipment failure.	Machine Learning (SVM)	Accuracy (95%)
El-Sayed et al. [8]	Google trace CMU OpenCloud LANL HPC Cluster	Design a job failure prediction model	Machine Learning (RF)	Precision (95%) & Recall (94%)
Shetty et al. [32]	Google trace	Predicting job termination status using resampling techniques	Machine Learning (XGboost classifier)	Precision (92%) & Recall (94.8%)
Proposed Model	Google trace Mustang Trinity	Design a failure prediction model	Machine Learning (RF, DTs, NB, QDA)	For Google trace Accuracy (99%) Precision (99%)

**Table 2:** Basic description and characteristics of the clusters having traces in the Atlas repository and Google cluster traces.

Dataset	Nodes	Sample Size	Features	Failed Ratio (%)	Length
Google Cluster	12,550	28,546,501	11	36.2	29 days
LANL Mustang	1600	2,113,175	9	7.2	5 years
LANL Trinity	9408	20,277	14	16.5	3 months

**Table 3:** Google trace overview.

Trace Characteristic	Value
Total number of users	933 users
Submitted jobs	676,975 jobs
Scheduling jobs	676,967 jobs
Finished jobs	386,218 jobs
Failed jobs	10,133 jobs
Killed jobs	272,609 jobs
Evict jobs	22 jobs
Lost jobs	16 jobs
Submitted tasks	48,330,301 tasks
Scheduling tasks	47,306,307 tasks
Finished tasks	18,187,970 tasks
Failed tasks	13,828,583 tasks
Killed tasks	10,337,327 tasks
Evict tasks	5,864,223 tasks
Lost tasks	8754 tasks

**Table 4:** Training and testing time and accuracy for all applied ML classifiers.

		DTs	RF	KNN	NB	Gradient Boosting	XGBoost
**Google (29 days)**	Training time	53.8	247.6	45.7	5.2	2093.8	2180.5
	Testing time	1.03	11	–	1	10.9	20.5
	Accuracy	98	98	–	78	96	96
**Google (7 days)**	Training time	13.23	75.7	5.6	0.8	344.46	182.1
	Testing time	0.16	1.9	2114.3	0.2	1.3	3.1
	Accuracy	98	98	97	92	97	97
**Mustang**	Training time	4.8	11.5	2.9	0.16	67.7	37.8
	Testing time	0.03	0.3	5.8	0.06	0.3	0.3
	Accuracy	99	99	95	86	97	97
**Trinity**	Training time	0.08	0.21	0.05	0.01	0.84	0.21
	Testing time	0.0009	0.007	0.16	0.001	0.007	0.007
	Accuracy	96	96	92	65	94	93

**Table 5:** Evaluated results of various feature selection methods.

		Random Forest					Decision Trees				
		Prec.	Rec.	F1-score	Train. (t)	Test. (t)	Prec.	Rec.	F1-score	Train. (t)	Test. (t)
**Google**	SelectKBest	98%	97%	97%	2683	39.5	97%	97%	97%	491.3	4.17
	Feature Importance	95%	93%	94%	2924	60.40	93%	93%	93%	518.3	7.9
	RFE	99%	99%	99%	2812	36.43	99%	99%	99%	542.18	3.39
**Trinity**	SelectKBest	72%	65%	69%	0.27	0.008	72%	69%	70%	0.05	0.0008
	Feature Importance	90%	88%	90%	0.38	0.02	89%	87%	88%	0.07	0.001
	RFE	85%	81%	83%	0.58	0.09	84%	83%	84%	0.09	0.008
**Mustang**	SelectKBest	94%	94%	94%	9.66	0.32	92%	93%	93%	0.9	0.03
	Feature Importance	95%	94%	95%	10.3	0.56	94%	94%	93%	1.2	0.09
	RFE	94%	93%	93%	11.1	0.73	93%	93%	93%	1.4	0.12

## Data Availability

Not applicable.
